# Complete Genome Sequence of *Floricoccus penangensis* ML061-4 Isolated from Assam Tea Leaf [*Camellia sinensis* var. *assamica* (J.W.Mast.) Kitam.]

**DOI:** 10.7150/jgen.83521

**Published:** 2023-07-16

**Authors:** Patthanasak Rungsirivanich, Elvina Parlindungan, Jennifer Mahony, Ian O'Neill, Brian McDonnell, Francesca Bottacini, Witsanu Supandee, Narumol Thongwai, Douwe van Sinderen

**Affiliations:** 1 Department of Biology, Faculty of Science, Chiang Mai University, Chiang Mai, Thailand.; 2 Graduate School, Chiang Mai University, Chiang Mai, Thailand.; 3 Community Development Department, Ministry of Interior, Bangkok, Thailand.; 4 School of Microbiology, University College Cork, Cork, Ireland.; 5 APC Microbiome Ireland, University College Cork, Cork, Ireland.; 6 Biological Sciences and ADAPT Research Centre, Munster Technological University, Cork, Ireland.; 7 Darunsikkhalai School, King Mongkut's University of Technology Thonburi, Bangkok, Thailand.; 8 Research Center in Bioresources for Agriculture, Industry and Medicine, Chiang Mai University, Chiang Mai, Thailand.

## Abstract

*Floricoccus penangensis* is a Gram-positive coccoid organism that is a member of the lactic acid bacteria. *F. penangensis* ML061-4 was originally isolated from the surface of an Assam tea leaf, and its genome is herein shown to contain gene clusters predicted to be involved in complex carbohydrate metabolism and biosynthesis of secondary metabolites.

*Floricoccus* is a component genus of the lactic acid bacteria (LAB), and has been classified as a member of the family *Streptococcaceae* 1,2. The genus *Floricoccus* was first described by Chuah *et al*. 1 and comprises two species i.e., *Floricoccus penangensis* and *Floricoccus tropicus.*

The Pacific Biosciences (PacBio) sequencing platform which is based on single-molecule real-time sequencing offers long read lengths and facilitates the improved assembly of genomes 3**.** A previous study by Chuah *et al*. 1 presented the genome sequences of *F*.* penangensis* and *F*.* tropicus* strains which were determined using an Illumina sequencing platform.

*F*.* penangensis* ML061-4 was originally isolated from the surface of an Assam tea leaf in the Sakat sub-district of Pua district, Nan province, Thailand (19°15′53.62″N, 101°0′30.22″E). Briefly, four square centimeters of the fresh leaf was swabbed using a sterile cotton swab moistened with 0.85% (v/w) NaCl (Sigma-Aldrich, MO, U.S.A.), which was then plated by streaking on tryptic soy agar (TSA; Merck, Darmstadt, Germany). The plate was incubated at 37°C for 24 h. A single colony of this strain was then re-streaked on TSA to obtain a pure culture 4. ML061-4 was routinely cultivated in M17 broth (Oxoid, Basingstoke, England) supplemented with 0.5% (w/v) glucose (GM17; Sigma-Aldrich) at 30°C for 24 h 5. Genomic DNA of *F*.* penangensis* ML061-4 was prepared using a NucleoBond DNA extraction kit (Macherey-Nagel, Dueren, Germany), and genome sequencing was carried out using a Pacific Bioscience (PacBio) SMRT RSII sequencing platform (PacBio, Menlo Park, CA, United States). The library was prepared by the sequencing facility (Macrogen NGS Services, Seoul, South Korea) using the SMRTbell template prep kit 1.0, following the guide for the PacBio RS System and selecting for inserts of approximately 10-15 kb. Size selection was performed by the third-party sequencing provider using the BluePippin system. Approximately 56k subreads were obtained and the approximate subread *N*_50_ of these reads was 7.1 kb.

All quality controls including adapter removal, reads trimming, and quality control were performed by Macrogen using SMRT Analysis portal v2.3.0 (https://smrt-analysis.readthedocs.io/en/latest/SMRT-Analysis-Software-Installation-v2.3.0). Filtered subreads were assembled using the Hierarchical Genome Assembly Process (HGAP) pipeline with the method RS_Assembly.2 implemented in SMRT Analysis portal v2.3.0. Automatic annotation of predicted open reading frames (ORFs) was performed using NCBI's Prokaryotic Genome Annotation Pipeline (PGAP) v5.2 6 to assign annotation, using an E-value cut-off of 0.0001 for hits showing at least 50% of similarity across at least 50% of the sequence length) against a non-redundant protein database provided by the National Centre for Biotechnology Information (NCBI) portal. Functional prediction of genes and proteins was integrated using the Clusters of Orthologous Groups (COGs) 7,8 and protein family (Pfam) 9, respectively, as previously described by Martín *et al*. 10. Ribosomal RNA (rRNA) and transfer RNA (tRNA) genes were detected using RNAmmer v1.2 11 and tRNA-scanSE v2.0 12, respectively.

The assembled (at ~120× coverage) genome of *F*.* penangensis* ML061-4 is composed of a single contig of 2,159,127 bp, with a GC content of 33.2%, which is predicted to encode 2,134 genes. The circular genome map of ML061-4 is presented in Figure 1. The chromosome contains 19 rRNAs, 63 tRNAs, 3 ncRNAs, 16 pseudogenes and 2,049 protein-coding genes. The genome sequence was deposited in GenBank under accession number CP075561. Genome mapping of *F*.* penangensis* ML061-4 was evaluated using CGView online server v1.7 (http://cgview.ca/) 13.

## Data availability

Genome sequence of *F. penangensis* ML061-4 was deposited in GenBank under accession number CP075561. The single-molecule real-time raw reads were deposited in SRA under the accession number SRX17727612.

## Figures and Tables

**Figure 1:**
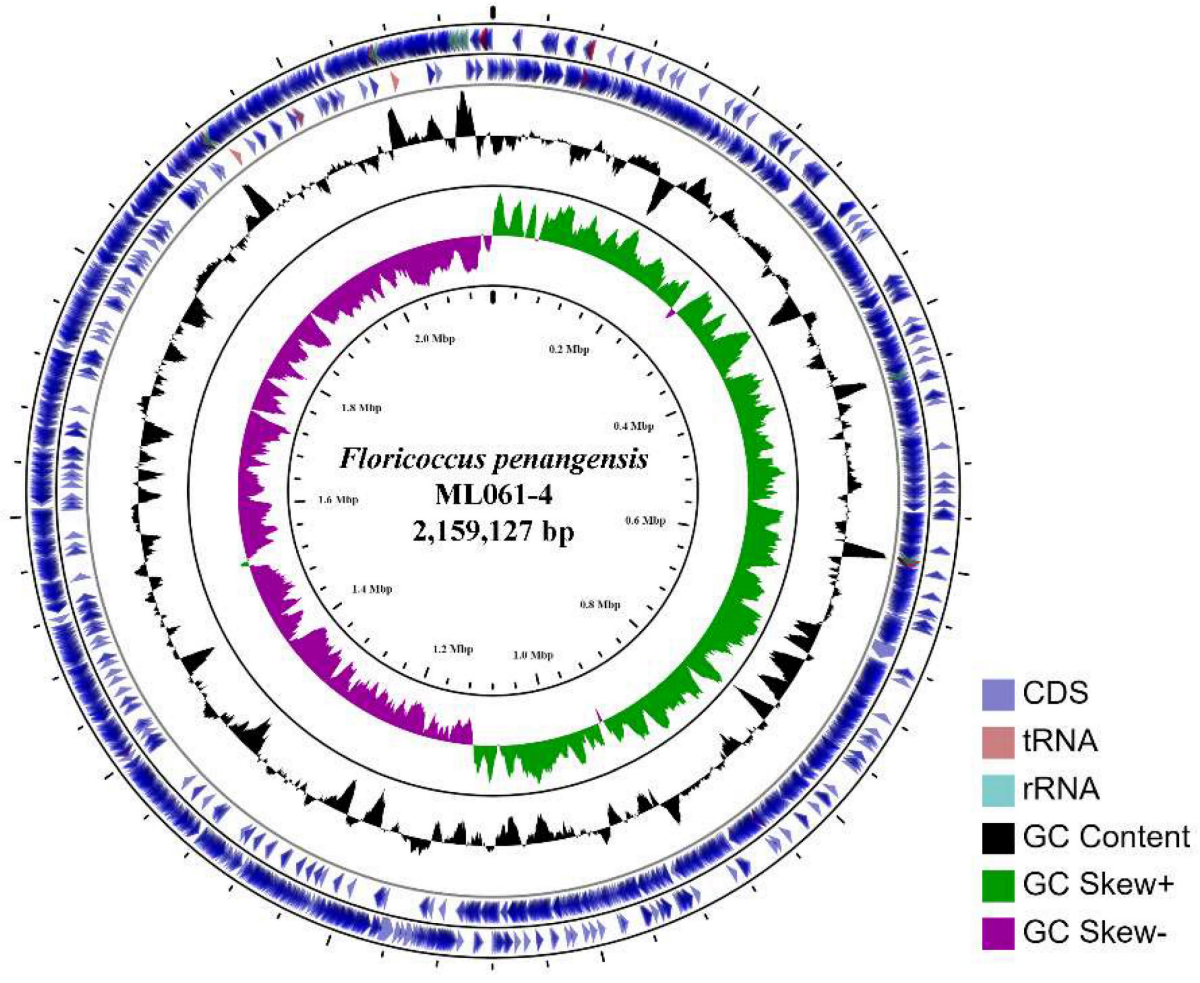
The circular chromosome of *F*.* penangensis* ML061-4 using CGView. From outside to inside are the coding sequence (CDS, blue), tRNA (red), rRNA (blue-green), GC content (black) and GC skew (green/purple).
